# The Sensitization Profile for Selected Food Allergens in Polish Children Assessed with the Use of a Precision Allergy Molecular Diagnostic Technique

**DOI:** 10.3390/ijms25020825

**Published:** 2024-01-09

**Authors:** Izabela Knyziak-Mędrzycka, Emilia Majsiak, Weronika Gromek, Danuta Kozłowska, Jakub Swadźba, Joanna Beata Bierła, Ryszard Kurzawa, Bożena Cukrowska

**Affiliations:** 1 Outpatient Allergology Clinic, the Children’s Memorial Health Institute, Aleja Dzieci Polskich 20, 04-730 Warsaw, Poland; i.knyziak-medrzycka@ipczd.pl; 2 Department of Health Promotion, Faculty Health of Sciences, Medical University of Lublin, Staszica 4/6, 20-081 Lublin, Poland; 3 Polish-Ukrainian Foundation of Medicine Development, Nałęczowska 14, 20-701 Lublin, Poland; weronikaa.gromek@gmail.com; 4 Diagnostyka S.A., Prof. M.Życzkowskiego 16, 31-864 Kraków, Poland; danuta.kozlowska@diag.pl (D.K.); jakub.swadzba@diag.pl (J.S.); 5 Department of Pathomorphology, the Children’s Memorial Health Institute, Aleja Dzieci Polskich 20, 04-730 Warsaw, Poland; j.bierla@ipczd.pl (J.B.B.); b.cukrowska@ipczd.pl (B.C.); 6 Department of Allergology and Pneumonology of the National Research Institute for Tuberculosis and Lung Diseases, Regional Branch in Rabka-Zdrój, Profesora Rudnika 3B, 34-700 Rabka-Zdrój, Poland; rkurzawa@igrabka.edu.pl

**Keywords:** sensitization profile, food allergens, allergy molecular diagnostic, molecular sensitization map

## Abstract

Individual populations show a variety of sensitization patterns, which may be associated with the geographic region, climate, dietary habits, or ways of preparing food. The purpose of this study was to comprehensively assess the food allergy sensitization profile in Polish children, particularly to eight food allergens (so-called “the Big 8”): cow milk, eggs, wheat, soybeans, fish, crustacean shellfish, tree nuts, and peanuts. To assess the prevalence and serum levels of specific immunoglobulins E (sIgE), we analyzed the results obtained from selected laboratories located in all regions of Poland that used the multiplex ALEX^®^ test in the period from 2019 to 2022. Results from 3715 children were obtained. The mean age of the study population was 7.0 years. The results were stratified by age: <12 months (3.63%), 1–5 years (39.54%), 6–13 years (46.32%), and 14–18 years (10.0%). The final analysis included the sIgE results obtained with 95 food extracts and 77 food allergen molecules. The highest rates of sIgE to food allergen extracts were found for peanut (29.20%), hazel (28.20%), and apple (23.60%), and those to allergenic molecules were found for the PR-10 family of molecules (Cor a 1.0401 (23.77%), Mal d 1 (22.37%), Ara h 8 (16.93%), and globulin 7/8S (Ara h 1; 15.59%)). The lowest rates of sIgE reactivity to extracts were found for strawberry (0.40%), oregano (0.30%), and thornback ray (0.16%), and those to allergenic molecules were found for Mal d 2 (0.27%) (thaumatin-like protein, TLP), Ani s 1 (0.30%) (Kunitz-type serine protease inhibitor), and Che a 1 (0.43%) (Ole e 1 family). The rates of sensitization to storage proteins of the analyzed “the Big 8” molecules decreased significantly (*p* < 0.05) with age. Conversely, the rates of sensitization to PR-10 family proteins increased significantly with age. The three most common allergens in Poland, regardless of whether IgE was assayed against extracts or molecules of food allergens, were peanut, hazel, and apple (in different order depending on the ranking). A detailed analysis of sensitization to the extracts and molecules of main food allergens based on the results of a multiplex ALEX^®^ test demonstrated the sensitization profile in Polish children (including molecular sensitization, particularly the “the Big 8” food allergen molecules), which shows considerable differences in comparison with those in other countries. Serum sIgE analysis of children from all regions of Poland revealed a food allergen molecular sensitization profile that changes with age.

## 1. Introduction

Food allergies are an increasingly common problem. There is a growing body of evidence showing that food allergies are on the rise [1]. Geographic and temporal differences in allergy prevalence reported so far indicate the role of environmental factors in allergy development [2,3,4]. 

Molecular diagnostics allows us to identify specific proteins to which a patient is allergic. This enables us to manage each patient’s allergy on an individual basis. By understanding the characteristics of the described proteins, we can predict the symptoms a patient may exhibit. An example is an allergy to profilins and polcalcins, which generally result in mild clinical symptoms. On the other hand, reserve proteins such as oleosins, nsLTP, tropomyosin, or parvalbumins can lead to anaphylaxis and even the death of the patient. Evaluating molecular sensitization helps better understand and track the allergic march that takes place in a given population and determine the most common allergenic molecules [5,6]. Molecular allergy diagnostics helps specify whether allergy symptoms are due to primary sensitization or cross-reactivity. Molecular sensitization assessment is a tool used to determine representative sensitization patterns in specific groups of patients. Such tests help develop diagnostic and therapeutic algorithms in early childhood. 

Different populations have various sensitization patterns. This was demonstrated in the year 2023 in a multi-center (Sweden, Norway, the Netherlands, the United Kingdom, Germany, Italy, Spain) study by Kiewiet et al., who assessed the specific immunoglobulin E (sIgE)-reactivity profile based on allergenic molecules in children (with an ImmunoCAP ISAC test, Phadia Austria GmbH, part of Thermo Fisher Scientific ImmunoDiagnostics, Vienna, Austria) [7]. The authors observed regional, exposome- and climate-dependent differences in molecular IgE-reactivity profiles in Northern, Western, Central, and Southern Europe, which may be a molecular basis for precision medicine-based approaches to allergy management and prevention.

Earlier studies on molecular sIgE reactivity profiles conducted in cohorts from individual countries (e.g., the United Kingdom, Germany, and Italy) also showed various patterns of molecular sensitization [8,9,10]. The differences in patterns may occur even within one country, as demonstrated in a study conducted in various regions of France [11]. That study showed significant differences in molecular sensitization profiles based on the differences in exposure to the given allergens, mainly as a result of the differences in the plants growing in different regions of the country. Likewise, in Kiewiet’s study, the exposome (primarily the climate) seemed to be an important factor affecting the differences in sIgE profiles observed between the cities of Rome and Bologna, Italy, and Sabadell and Gipuzkoa, Spain [7]. 

The purpose of our study was to comprehensively assess pediatric sensitization profiles to food allergens, focusing particularly on eight food allergens (so-called “the Big 8”): cow milk, eggs, wheat, soybeans, fish, crustacean shellfish, tree nuts, and peanuts. Diagnostic tests were conducted with the use of precision molecular diagnostics, and the obtained data helped create an age-dependent sensitization profile of Polish children. Creating such a sensitization profile helps identify the most common allergens, determine their characteristics and the risk of anaphylaxis, and draw the conclusions necessary to manage allergy problems in entire populations. Our study provides a comprehensive review of IgE sensitization profiles based on both molecular and extract analyses of food allergens in children and adolescents living in Poland. To the best of our knowledge, this is the first analysis of the results of multiplex third-generation tests conducted on children from various regions of Poland.

## 2. Results

### 2.1. Subjects’ Characteristics

We analyzed the test results of 3715 children who were diagnosed in search of allergic causes of their various symptoms in several Polish laboratories in the period from 2019 to 2022. More than half of the analyzed group (58.0%) were boys. The mean subject age was 7.0 years. The youngest subject was 2 months old, and the oldest was 17.3 years old. The largest age groups were 6–13 year-olds (46.32%) and 1–5 year-olds (39.54%). A detailed analysis is in Table 1.

Out of the collected test results, 572 results were obtained with the ALEX^®^ test (assessing 156 extracts and 126 allergen molecules), and 3143 results were obtained with the ALEX^®2^ test (assessing 117 extracts and 178 allergen molecules). A total of 1,088,489 individual sIgE tests were obtained and analyzed, including 631,526 sIgE tests (58.0%) for allergen molecules and 456,963 sIgE tests (42.0%) for allergen extracts. Three molecules were excluded from the overall allergen molecule analysis. Two of those were excluded because they were a mix of molecules from different groups, which made it impossible to assign them to a specific family of molecules—these were a mix of strawberry molecules (Fra a 1 + Fra a 3, mix) and a mix of cod molecules (Gad m 2 + Gad m 3)—and the third one was hazelnut molecule Cor a 11, which was due to a data transfer error resulting in only 197 test results being available for this molecule.

Ultimately, our analysis included sIgE test results for 95 food allergen extracts and 77 food allergen molecules. The list of all analyzed allergens has been made available as supplementary data (Tables S1 and S2).

### 2.2. Sensitization Profile to Selected Food Allergens Based on Allergen Extracts

Table 2 in Part A presents the ranking of selected allergen extracts, namely those against which the proportion of sIgE exceeded 10%. All data have been included in supplementary data (Table S1). Out of 3715 sIgE results obtained with the ALEX^®^ test for 95 allergen extracts, the most common food allergens were peanuts (29.20%), hazelnuts (28.20%), and apples (23.60%). Conversely, the allergens that yielded the lowest proportion of positive tests were strawberry (0.40%), oregano (0.30%), and thornback ray (0.16%). The highest observed mean sIgE levels were for cow milk (9.86 kU_A_/L), shrimp (8.55 kU_A_/L), and peanut (8.54 kU_A_/L), whereas the lowest mean sIgE levels were for lychee extract (0.49 kU_A_/L), mushroom (0.45 kU_A_/L), and strawberry (0.40 kU_A_/L).

Apart from peanut and hazelnut, all other “the Big 8” food allergens were also analyzed; these included hen eggs, cow milk, fish, wheat, soybeans, shellfish, and other tree nuts. Out of these other “the Big 8” allergens, hen egg white ranked the highest, taking 4th place, whereas egg yolk ranked 35th, with mean levels of 5.46 kU_A_/L and 3.26 kU_A_/L, respectively. Out of the 3715 evaluated children, 383 (10.31%) were positive for cow milk sIgE (with mean levels of 9.86 kU_A_/L) assessed with a cow milk extract, which placed this allergen at the top of the ranking for average sIgE concentration for extract-based sensitization.

Analysis of sIgE against fish allergens ranked cod at the 41st place (4.23%), Atlantic herring at the 55th place (2.67%), salmon at the 62nd place (1.99%), tuna at the 75th place (0.94%), Atlantic mackerel at the 76th place (0.92%), and thornback ray at the 85th place, with the mean sIgE levels of 7.08 kU_A_/L, 4.42 kU_A_/L, 3.63 kU_A_/L, 1.11 kU_A_/L, 0.92 kU_A_/L, and 0.78 kU_A_/L, respectively.

Common wheat and spelt wheat ranked 39th and 40th, with similar rates of 4.60% and 4.50%, respectively. The mean sIgE levels were 4.24 kU_A_/L against common wheat and 3.32 kU_A_/L against spelt wheat. sIgE against soybean extract was detected in 73 children (14.60%) at a mean level of 3.76 kU_A_/L, which made this allergen rank as the 7th most common positive sIgE test based on allergen extracts and in place of the 33rd in the analysis of IgE concentrations for food extracts.

Out of four crustacean shellfish extracts, the most common positive sIgE result was found for lobsters (2.21%), placing this allergen at the 60th rank with mean sIgE levels of 5.65 kU_A_/L. Crab ranked 69th with the mean sIgE levels of 8.38 kU_A_/L, and the extracts of various species of shrimp (Pan b and Lit s z 2) ranked 71st and 72nd, with the mean sIgE levels of 6.17 and 8.55 kU_A_/L, respectively. 

The test included six tree nut allergen extracts. Apart from hazel, which ranked 2nd, the remaining nuts, namely cashew, walnut, pecan, Brazil nut, and macadamia nut, ranked 5th, 9th, 11th, 19th, and 32nd, respectively, with mean sIgE levels of 7.44 kU_A_/L, 5.78 kU_A_/L, 6.64 kU_A_/L, 4.06 kU_A_/L, and 5.47 kU_A_/L, respectively. Table 2 presents the ranking of sIgE in allergen extracts for food allergens, with rates of more than 10%.

### 2.3. Sensitization Profile for Selected Food Allergens Based on Allergen Molecules

Table 2 presents those 17 out of all 77 tested food allergen molecules that yielded positive sIgE results in over 10% of the analyzed population. The entire ranking of sIgE rates in response to all analyzed allergen molecules has been attached as supplementary data (Table S2). Out of the 3715 sIgE results obtained with the ALEX^®^ test, the most common food allergen molecules yielding a positive sIgE response were those from the PR-10 family. Molecules from this family constituted six out of ten food allergen molecules with the highest sIgE rates; these six molecules were: Cor a 1.0401 (23.77%), Mal d 1 (22.37%), Ara h 8 (16.93%), Gly m 4 (15.18%), Api g 1 (15.07%), and Dau c 1 (13.76%). These six molecules ranked 1st, 2nd, 3rd, 5th, 6th, and 7th in the overall ranking of allergen molecules producing positive sIgE responses in the greatest proportion of the study population. One molecule from outside the PR-10 family that ranked in the top five positions was Ara h 1 (globulin 7/8S) (15.59%), a peanut molecule. The lowest proportion of the population showed sIgE to Mal d 2 (0.27%) (TLP), Ani s 1 (0.30%) (Kunitz-type serine protease inhibitor), and Che a 1 (0.43%) (Ole e 1 family). 

We analyzed the mean levels of sIgE (kU_A_/L) against the individual allergen molecules. The highest observed sIgE levels were against Ara h 2 (15.92 kU_A_/L) (albumin 2S) and Ara h 6 (14.43 kU_A_/L) (albumin 2S), Jug r 1 (14.19 kU_A_/L) (globulin 7/8S), Cuc m 2 (12.92 kU_A_/L) (profilin), Thu a 1 (12.26 kU_A_/L) (β-parvalbumin), Cor a 1.0401 (12.22 kU_A_/L) (PR-10), Clu h 1 (12.07 kU_A_/L) (β-parvalbumin), Ana o 3 (11.93 kU_A_/L) (globulin 11S), Pis v 1 (11.50 kU_A_/L) (albumin 2S), and Sal s 1 (11.44 kU_A_/L) (β-parvalbumin) molecules. The lowest observed mean sIgE levels were against Ara h 15 (1.00 kU_A_/L) (oleosin), Mal d 2 (0.99 kU_A_/L) (TLP), and Ani s 1 (0.78 kU_A_/L) (Kunitz-type serine protease inhibitor) molecules.

### 2.4. sIgE against “the Big 8” Food Allergen Molecules Stratified by Age

**Figure 1:**
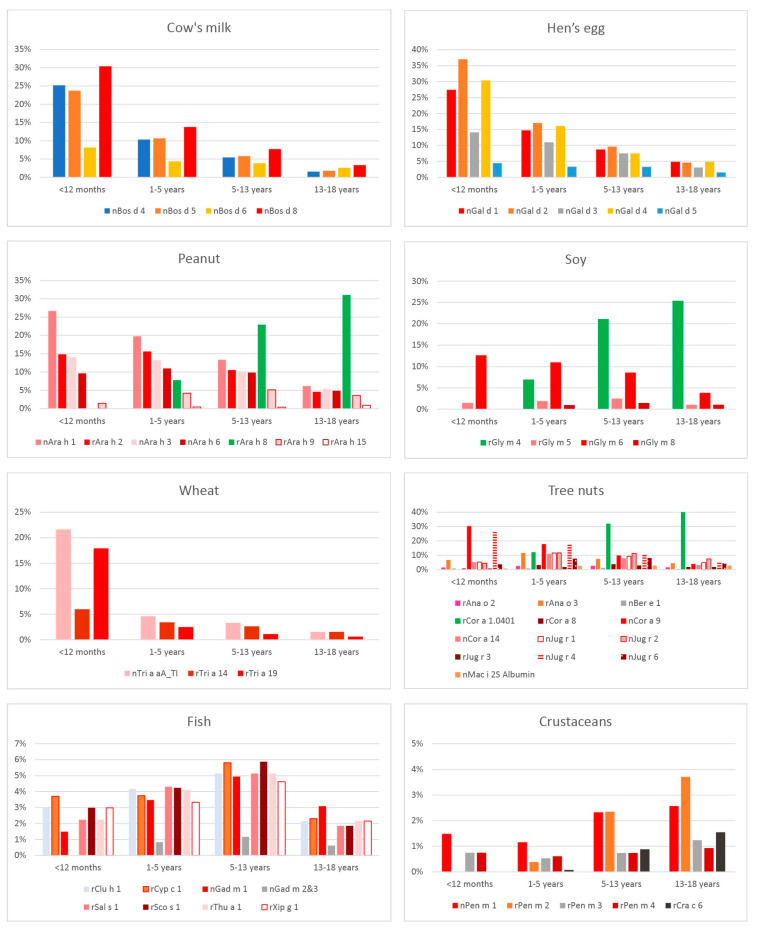
The sIgE rates against the “the Big 8” allergen molecules stratified by age. Blue columns represent serum albumins, green columns represent proteins of the PR-10 family, and red columns (of various shades and patterns) represent molecules associated with a high risk of anaphylactic shock (the remaining colors, even repeated ones, do not indicate any relationships between them).

Figure 1 shows the proportion of the evaluated individuals with sIgE against the “the Big 8” food allergens: cow milk, eggs, peanuts, soybeans, wheat, fish, crustaceans, and tree nuts.

Children from the youngest evaluated age range (<1 year of age) showed the highest rates of positive sIgE results against all cow milk molecules, with casein (Bos d 8) yielding the highest proportion of positive tests (30.37%). The age group with the lowest rates of positive sIgE against all analyzed cow milk molecules was the 13–18 year-olds. Molecules Bos d 4, Bos d 5, and Bos d 8 showed a significant decrease in positive sIgE rates with age in all analyzed age groups (*p* < 0.05). (All statistical differences with respect to the discussed number of children with sIgE against “the Big 8” food allergen molecules stratified by age can be found in “Table S3: sIgE against “the Big 8” food allergen molecules stratified by age”). Another molecule, Bos d 6, showed a similar decrease with age; however, only the difference between the <12 month-olds and 1–5 year-olds reached statistical significance.

A detailed analysis showed the egg molecule that yielded the highest rates of positive sIgE results was Gal d 2 (*n* = 483; 13.00%), with mean sIgE levels of 4.80 kU_A_/L, followed by egg white molecules Gal d 4 (425; 11.44%), Gal d 1 (*n* = 422; 11.36%), and Gal d 3 (321; 8.64%). In the overall ranking of allergen molecules producing positive sIgE results, these egg molecules ranked 10th, 13th, 14th, and 22nd, respectively. The highest mean levels of sIgE against egg molecules were observed for Gal d 1 (6.36 kU_A_/L). The lowest rates of sIgE were observed in response to the Gal d 5 (*n* = 118; 3.18%) egg yolk molecule; this allergen component also yielded the lowest mean sIgE levels of 3.07 kU_A_/L. Among the analyzed food molecules, egg molecules Gal d 2, Gal d 4, Gal d 1, Gal d 3, and Gal d 5 based on average sIgE concentration ranked 47th, 38th, 33rd, 46th, and 61st place, respectively. The age group with the highest rates of sIgE against egg molecules was the group of infants, whereas the lowest sIgE rates were observed in 13–18 year-olds. Analysis of positive sIgE results for Gal d 1, Gal d 2, Gal d 3, and Gal d 4 of eggs showed a significant decrease in positive reactions with age (*p* < 0.05). This decrease was also observed, but did not reach statistical significance, for Gal d 3 and Gal d 5 between the groups of <12 month-olds and 1–5 year-olds and for Gal d 4 between the groups of 5–13 year-olds and 13–18 year-olds. 

Peanut molecules ranked 3rd (Ara h 8), 4th (Ara h 1), 12th (Ara h 2), 15th (Ara h 3), 18th (Ara h 6), 39th (Ara h 9), and 72nd (Ara h 15) in the overall rates of sIgE to allergen molecules. Analysis of mean sIgE levels for all analyzed food molecules showed the highest levels of antibodies to Ara h 2 (15.92 kU_A_/L), followed by Ara h 6 (14.43 kU_A_/L). The lowest mean sIgE levels were observed for Ara h 15 (1.00 kU_A_/L), which ranked third out of the mean sIgE levels for food allergen molecules. The number of positive sIgE results for Ara h 1 decreased with age, whereas those for Ara h 8 were absent in <12 month-olds, were first detected in 1–5 year-olds, and increased in consecutive older age groups. There was a significant (*p* < 0.05) decrease in the number of positive sIgE results for Ara h 1, Ara h 2, and Ara h 3 molecules between 1–5 year-olds and 5–13 year-olds and between 5–13 year-olds and 13–18 year-olds. Conversely, there was a significant (*p* < 0.05) increase in positive sIgE tests to Ara h 8 with age between each of the consecutive age groups.

The ranking of food allergen molecules with the highest positive sIgE test results revealed Gly m 4 to be in 5th place (15.18%), with mean levels of 9.91 kU_A_/L. Although these antibodies were not detected in under 12 month-olds, their levels increased considerably in subsequent age groups (ages 1–5, 5–13, and 13–18 years). This increase was statistically significant (*p* < 0.05) between the groups of <12 month-olds and 1–5 year-olds and between the groups of 1–5 year-olds and 5–13 year-olds. The Gly m 6 molecule produced the 20th highest sIgE response rates of 9.18%, with a mean sIgE level of 4.63 kU_A_/L; and the Gly m 5 molecule ranked 58th (2.07%), with a mean level of sIgE of 1.92 kU_A_/L. Out of soybean molecules, the one that produced the lowest rates of sIgE (1.16%) was Gly m 8 (66th place), with a mean sIgE level of 2.39 kU_A_/L. The number of positive sIgE results for the Gly m 6 protein decreased with age, with significant differences between the age groups of 1–5 and 5–13 years (*p* = 0.025) and between the age groups of 5–13 and 13–18 years (*p* = 0.001).

The wheat molecule that produced the highest sIgE response rates (4.45%) in the overall ranking of food molecules was Tri a A_TI (40th place), with a mean sIgE level of 5.78 kU_A_/L. The next wheat molecule, Tri a 14, ranked 50th, producing 2.99% positive sIgE responses with a mean sIgE level of 6.52 kU_A_/L. The final wheat molecule, Tri a 19, ranked 56th, with 2.35% positive sIgE responses and a mean sIgE level of 4.02 kU_A_/L. Analysis of sIgE to wheat allergen molecules showed the highest positive sIgE levels to all evaluated allergen molecules in the <12 month age group, and then the levels of sIgE decreased with age for all analyzed wheat molecules, with the decrease reaching statistical significance for Tri a aA_TI between the groups of <12 month-olds and 1–5 year-olds (*p* = 0.001) and for Tri a 19 between <12 month-olds and 1–5 year-olds (<0.001) and between 1–5 year-olds and 5–13 year-olds (*p* = 0.006).

In terms of sIgE levels to fish molecules, the thornback ray molecule Raj c-parvalbumin ranked last, producing 0.64% positive responses, whereas the highest sIgE rates were due to the Sco s 1 molecule of Atlantic mackerel. The highest mean sIgE levels of fish allergen molecules were produced by Thu a 1 (12.26 kU_A_/L). The sIgE rate-to-age analysis of fish allergen molecules showed the highest sIgE levels of all evaluated fish molecules in the 5–13 year age group.

An inverse tendency was observed with shrimp molecules, particularly Pen m 2 and Cra c 6. All evaluated shrimp molecules produced the highest rates of positive results in the age group of 13–18 year-olds. The ranking of sIgE to allergen molecules showed shrimp molecules taking the 60th (Pen m 1), 63rd (Pen m 2), 69th (Pen m 3 and Pen m 4, ex aequo), and 71st (Cra c 6) places. The highest observed mean sIgE levels were for Pen m 1 (9.98 kU_A_/L) and Cra c 6 (9.65 kU_A_/L), and the lowest were for Pen m 3 (3.16 kU_A_/L). The differences in sIgE levels between Pen m 1 (*p* = 0.013) and Pen m 2 (*p* < 0.001) were statistically significant between the age groups of 1–5 year-olds and 5–13 year-olds.

A detailed analysis of tree nut allergens showed the allergen molecule most commonly producing a positive sIgE response was the Cor a 1.0401 molecule of hazelnut, with a mean sIgE level of 12.22 kU_A_/L. Among the 30 food allergen molecules most commonly producing a positive sIgE response, there were as many as eight molecules of various tree nuts (Cor a 1.0401, Jug r 4, Cor a 9, Jug r 2, Jug r 1, Ana o 3, Cor a 14, and Jug r 6). The highest sIgE levels were due to the hazelnut molecule Jug r 1 (14.19 kU_A_/L), which was characterized as producing the 3rd highest mean sIgE levels in the overall ranking of all allergen molecules. There was a statistically significant increase in the rates of positive sIgE responses to Cor a 0401 and a decrease in sIgE responses to Cor a 9 in all evaluated groups. The ranking of positive sIgE responses to “the Big 8” food allergen molecules has been presented in Table 3, while Table S3 presents sIgE against “the Big 8” food allergen molecules stratified by age.

## 3. Materials and Methods

### 3.1. Subjects and Study Design

The rates and levels of sIgE in Polish children were determined retrospectively based on blood test results obtained from selected laboratories located in various regions of Poland. The data were obtained from the Immunology Laboratory, Department of Pathomorphology of the Children’s Memorial Health Institute in Warsaw, Poland, from the National Research Institute for Tuberculosis and Lung Diseases, Regional Branch in Rabka-Zdrój, Poland, and the network of Diagnostyka S.A. laboratories, conducting tests in all parts of Poland. The analysis of the prevalence of sIgE was based on the results of the ALEX^®^ test (Macro Array Diagnostics GmbH, Vienna, Austria) of all children (0 to 18 years old) who were diagnosed at the above-mentioned Polish institutions in the period between 2019 and 2022. The obtained data were devoid of information that would allow the patient’s identity to be revealed. Apart from the results of the ALEX^®^ test, only the age and sex of each patient were included.

The analysis of the data was conducted as part of the Children’s Memorial Health Institute internal project No. S168/2018 (principal investigator B.C.). The study was approved by the local ethics committees at the Children’s Memorial Health Institute (approval No. 50/KBE/2018 of 21 November 2018) and Medical University in Lublin (approval No. KE-0254/86/03/2023 of 30 March 2023).

### 3.2. Multiplex ALEX^®^ Test

The ALEX^®^ test is a third-generation multiplex test for measuring the levels of sIgE against allergen extracts and molecular components simultaneously. In 2017, the ALEX^®^ test allowed for measuring sIgE levels for 282 allergen components from 67 sources: 156 extracts and 126 molecular components. In 2019, the composition of the ALEX^®^ test was altered: several allergens (mainly extracts) were removed, and new allergens (mainly allergen molecules) were added. The name of the test now also includes the numeral 2, which indicates the altered composition. Since the year 2019, ALEX^®2^ has helped determine sIgE levels against 295 allergens, including 117 extracts and 178 allergen molecules from various sources, such as foods, animals, plants, molds, and others. Therefore, some of the patients had ALEX^®^ and some had ALEX^®2^, which is important in terms of the number of tests for individual allergen extracts or molecular components (sIgE rates have been calculated with respect to the total number of tests conducted for the individual extract or molecule). For the purposes of this article, the name ALEX^®^ is used throughout both tests. A detailed analysis of the incidence of sIgE included molecules of “the Big 8” allergens, namely cow’s milk (nBos d 4, nBos d 5, nBos d 6, nBos d 8), hen egg (nGal d 1, nGal d 2, nGal d 3, nGal d 4, nGal d 5), wheat (nTri a aA_TI, r Tri a 14, nTri a Gliadin, rTri a 19), peanut (nAra h 1, rAra h 2, nAra h 3, nAra h 6, rAra h 8, rAra h9, rAra h 15), tree nut (rCor a 1.0401, nCor a 11, nJug r 4, nCor a 9, nJug r 2, nJug r 1, rAna o 3, nCor a 14, nJug r 6, rCor a 8, rAna o 2, rJug r 3, nBer e 1) fish (rSco s 1, rCyp c 1, rClu h 1, rSal s 1, rThu a 1, nGad m 1, rXip g 1, rRaj c Parvalbumin) and peanut–shellfish (nPen m 1, rPen m 2, rPen m 3, rPen m 4, rCra c 6). In accordance with the normal sIgE ranges provided by the manufacturer, a test was considered positive if its result was ≥0.3 kilounits of allergen sIgE per liter (kU_A_/L). The results were exported from MADx Raptor Software to Excel spreadsheets. The results obtained with these tests are quantitative and are expressed in units of IgE response (kU_A_/L).

### 3.3. Statistical Analysis

Statistical analysis was performed using IBM^®^ SPSS^®^ 24.0.0.0. Statistics. Chi-squared tests were used to define statistically significant correlations between qualitative variables. To measure the correlation between variables, Spearman’s rank correlation coefficient was used. Continuous variables were summarized using mean values, while variability around mean values was reported in terms of standard deviations (SD). A *p*-value < 0.05 was considered statistically significant.

## 4. Discussion

The three most common allergens in Poland, regardless of whether IgE was assayed against extracts or molecules of food allergens, were peanut, hazel, and apple (in different order depending on the ranking).

Allergen extracts

There have been many sensitization assessments based on allergen extracts conducted over the years [3,4]. In our study, the most common food allergens detected with the use of allergen extracts were peanut, hazelnut, and apple. On the other hand, the allergen extracts producing the lowest rates of positive sIgE tests were strawberry, oregano, and thornback ray. The EuroPrevall study [3] showed geographic differences in the prevalence of food sensitization and food allergy in school-age children (7–10 years) across many European cities (Zurich, Madrid, Athens, Utrecht, Vilnius, Lodz, and Reykjavik). As part of that study, 24 foods considered to be the most sensitizing to children or most commonly consumed in the countries involved were selected for sIgE analysis. These were eggs, cow milk, fish, shrimp, peanuts, hazelnuts, walnuts, peach, apple, kiwi, melon, banana, tomato, celery, carrot, corn, lentils, soybeans, wheat, buckwheat, sesame, mustard, sunflower, and poppy seed. None of the cities involved in the EuroPrevall study reported peanuts to be among the top three sensitizing allergens, whereas in our study peanuts ranked first. The EuroPrevall study results, not unlike ours, showed hazelnuts to be among the allergens with the highest sIgE rates. In our study, it ranked second, and in the EuroPrevall study, it ranked first in Lodz, second in Utrecht, and third in Zurich and Vilnius. One of the most common food allergens was banana, which ranked first in Zurich and Madrid, second in Athens and Vilnius, third in Utrecht and Reykjavik, and fourth in Lodz. In our study, bananas ranked as the 73rd most common food allergen out of all evaluated allergen extracts [3]. 

Apple, which ranked third in our analysis, did not rank among the top three sensitizing allergens in any of the cities taking part in the EuroPrevall study. In our study, the rate of sensitization to apples was 23.60%, whereas in the EuroPrevall study, it ranged from 2.05% to 11.95% (depending on the city). The rates of cow milk and egg sensitization were also higher in our study than those of shrimp (with the latter common in the Mediterranean and in Reykjavik). Despite the fact that peach allergens ranked second in Lodz (which is located in Poland—the country where our study was conducted) and Madrid, they ranked as the 26th most common cause of sensitization in our study [3]. 

In summary, our analysis, which was based on a very broad spectrum of allergen extracts, showed considerable differences in the sensitization profiles of Polish children in comparison with those reported by other authors.

Allergen molecules

Expanding the diagnostics to include allergen molecules allows for a more detailed assessment of the sensitization profile. Molecular diagnostics helps detect and quantify serum sIgE for a specific allergen molecule. An allergen extract is a collection of multiple proteins of a three-dimensional structure composed of polypeptide chains, namely allergen molecules, which are considered to be allergens proper in this type of diagnostic. Since various allergen molecules are of varied diagnostic and clinical significance, molecular diagnostics offers a much more accurate assessment of sensitization and its importance for the patient, along with the natural course of the given allergic condition [5]. Allergen component-based diagnostics increases assessment specificity, which helps assess primary sensitization, and increases test sensitivity, particularly if the allergens present in the extract are of insufficient quantity or are destroyed during the process of preparing the extract for analysis [6]. 

Our analysis of the rates of sIgE in food allergen molecules showed panallergen proteins of the PR-10 subfamily to produce the highest sIgE rates. The top 10 food allergen molecules producing the highest sIgE responses included as many as six proteins from this subfamily; these were Cor a 1.0401, Mal d 1, Ara h 8, Gly m 4, Api g 1, and Dau c 1. Allergy to these molecules is likely to be a result of cross-reactivity to a primary birch allergy (Bet v 1 molecule) [12]. A multicenter study by Kiewiet et al. showed that cross-reacting proteins of the PR-10 family, such as Cor a 1.0401, Mal d 1, and Pru p 1, are among the molecules most commonly bound by sIgE in cohorts with high rates of allergy to Bet v 1, due precisely to cross-reactivity [7]. Likewise, the EuroPrevall study showed that a hazelnut allergy, one of the most common allergies in various European cities, may be associated with cross-reactivity to birch pollens, which are common in Central/Northern Europe. One explanation for such high rates of proteins from the PR-10 family among the top ten allergen molecules may be provided by Westman et al. [13]. Those authors reported sIgE reactivity to PR-10 proteins to have a hierarchic intrarelationship, with Bet v 1 producing the highest sIgE reactivity and the following allergens producing gradually decreasing reactivity: Bet v 1 > Mal d 1 > Cor a 1.0401 > Ara h 8 > Pru p 1 > Aln g 1 > Api g 1 > Act d 8 > Gly m 4. In our study, the order of sIgE reactivity was only slightly different and was as follows: Bet v 1 > Cor a 1.0401 > Mal d 1 > Ara h 8 > Gly m 4 > Api g 1 > Dau c 1. 

In summary, our analysis of sIgE reactivity profiles to allergen molecules showed the highest rates of sIgE to PR-10 proteins, which may be associated with environmental exposure to birch pollen in Poland (birch constitutes 7% of the 647,000 hectares of total forested area in Poland), as reported in the Forestry Almanac of 2021 issued by the Central Statistical Office [14]. 

sIgE reactivity to “the Big 8” molecules stratified by age

Cow’s milk is one of the foods most commonly consumed by children. Therefore, sensitivity and allergy to cow milk proteins are the subject of a number of studies [7,15]. Our analysis showed the rates of sIgE reactivity to all cow milk proteins to be the highest in <12 month-olds and the lowest in 13–18 year-olds. Our analysis showed the highest rates of sIgE reactivity (10.47%) to cow milk casein (Bos d 8) and the lowest (4.06%) to Bos d 6. In contrast, Kiewiet et al., who assessed various European pediatric cohorts with the ImmunoCap ISAC method in a multicenter study, demonstrated the rates of allergy to cow milk allergen molecules to be much lower, in most cases under 1% (with the exception of one Spanish group from Sabadell, where sIgE rates were 1.4–2.0%) [7]. In a study conducted in a Swedish cohort, the rates of sIgE reactivity to Bos d 4, Bos d 5, Bos d 6, and Bos d 8 increased with age [7], whereas analysis of our study cohort showed a significant decrease in the rates of positive sIgE responses to Bos d 4, Bos d 5, and Bos d 8 molecules with increasing age.

As with cow milk proteins, our analysis of sIgE reactivity to egg molecules showed a significant decrease in positive test results for Gal d 1, Gal d 2, Gal d 3, and Gal d 4 molecules with increasing age. In our study, the allergy rates to ovalbumin were 13.0% and to ovomucoid 11.4%. Interestingly, in the study by Kiewiet et al., the thermally stable egg allergen Gal d 1 was the most prominent in the Dutch study population over 1 year of age, whereas, in our study population, the highest rates of this allergen were observed in the under-12 month-olds [7]. Like in our study, Lin et al. reported sensitization rates to hen egg molecules to be decreasing with age in children with atopic dermatitis. The rates of serum sIgE to ovalbumin and ovomucoid in various age groups analyzed in that study were the highest in 0–2 year-olds and decreased with age [16].

The peanut molecules that showed the highest rates of sensitization in Polish children were Ara h 8 and Ara h 1, with an age-dependent sensitization profile. The rates of Ara h 8 sensitization increased significantly with age. Starting from absent sIgE (0.00%) to Ara h 8 in the group of <12 month-olds, subsequent age groups showed increasing rates of sIgE to reach the highest rates at 31.0% in the group of 13–18 year-olds. We believe that the increase in Ara h 8 sensitization rates with age observed in our cohort may be associated with sensitization to the birch Bet v 1 molecule. This is associated with the phenomenon of becoming sensitized to PR-10 proteins with increasing age, which may be related to environmental exposure to birch pollen. Studies conducted by other authors have shown considerable differences in the rates of sensitization to various peanut molecules, depending on the country. Patients with peanut allergy in the United States and Sweden were shown to have higher rates of allergen storage proteins Ara h 1–3 in comparison with those in patients from Spain, who showed higher rates of sensitization to lipid transfer protein Ara h 9 [17]. Kiewiet et al. demonstrated that Ara h 9 sensitization rates were higher in the cohort from southern Europe, like Spain (Gipuzkoa) and Italy (Rome), in comparison with those in the Swedish cohort [7]. In our study, the molecule with the highest sensitization rates in children up to 5 years old was Ara h 1 (26.7% in <12 month-olds and 19.7% in 1–5 year-olds). These results are partly consistent with those from studies conducted in English and Swedish populations, which showed Ara h 1 to be the molecule with the highest sensitization rates in 4 year-old children sensitized to peanuts. However, in contrast with Swedish studies, which showed a trend towards Ara h 1 sIgE rates increasing with age [7], our study showed the rates of sIgE not only to Ara h 1 but also to Ara h 2 and Ara h 3 to decrease with age. The differences in Ara h sensitization profiles may be due to the amounts consumed, the age of peanut introduction, dietary habits, and the different ways in which peanuts are prepared for consumption in various countries. The high peanut sensitization rates in Sweden, the United States, and other western countries are suspected to be a result of the fact that peanuts are most often consumed roasted (roasting increases the sensitizing potential of Ara h 1 and Ara h 2 molecules) or may expose proteins, such as Ara h 15 (oleosin), that would otherwise be enveloped in the fat that constitutes energy stores for the sprouting plants [18,19,20]. 

Our analysis of soybean-sIgE rates showed the Gly m 4 molecule to yield the highest positive sIgE rates of 15.2%. Similar results were obtained in a German cohort, where Gly m 4 was the most prevalent (10.5%) sensitizing soybean molecule [7]. In our study, Gly m 4 (as well as another PR-10 subfamily molecule, Ara h 8) produced no positive sIgE results in the youngest evaluated age group of up to 12 month-old infants; however, a statistically significant increase in the sIgE rates for this molecule was observed with increasing age. The observed increase in the rates of sensitization to Gly m 4 (as well as to Ara h 8) with age may be associated with the increase in the rates of birch allergen sensitization with age. Studies show that a majority of patients with birch allergy are sensitized to the main allergen of birch pollen (Bet v 1), the main allergen of apple (Mal d 1), and soybeans (Gly m 4) [12]. Extended analysis of a correlation between Bet v 1 and Gly m 4 sIgE rates in our study group revealed only 0.88% of children (5/564) with positive sIgE reactivity to Gly m 4 who had no positive sIgE reactivity to Bet v 1 (data not shown). Like us in our study, Westman et al. also observed a close correlation between sIgE reactivity to Gly m 4 and Bet v 1 and the number of other IgE-reactive PR-10 proteins. Those authors showed that 100% of children’s sIgE reactivity to Gly m 4 also had sIgE to Bet v 1, Mal d 1, and Cor a 1.0401 [13]. 

In our study, the wheat molecules with the highest sIgE rates were Tri a A_TI (4.45%), followed by Tri a 14 (2.99%), and Tri a 19 (2.35%). The highest rates of positive sIgE for all evaluated allergen molecules were observed in the <12 month-olds and the lowest ones in 13–18 year-olds. Kiewiet et al. reported sIgE rates ranging from 0.1% in the Swedish cohort to 0.4% in the Norwegian cohort, and no diminished sensitization to this molecule with age was observed. In the Swedish cohort, the groups of 4 year-olds, 7–12 year-olds, and 15–16-year-olds had a steady Tri a 14 sensitization rate of 0.1%. The Norwegian cohort showed sIgE levels to Tri a 19.0101 that were similar to those in our study, although they differed between age groups, with the highest sIgE rates of 3.3% in 15–16 year-olds and lower rates in 7–12 year-olds (3.0%) [7].

One of the most important fish proteins is parvalbumin. Our analysis of fish allergen molecule-sIgE rates with respect to age showed the highest rates of sIgE among all evaluated fish molecules found in the 5–13 year age group. The rates of sIgE in fish ß-parvalbumin ranged from 3.75% to 4.65% (with the lowest ß-parvalbumin sIgE rates for swordfish and the highest for Atlantic mackerel, carp, Atlantic herring, salmon, tuna, and cod). The ALEX^®^ test allows for testing two parvalbumin variants: α and β. Despite the high homology between α- and β-parvalbumin, the allergenicity of fish α-parvalbumin is generally considered to be very low and well tolerated. Nonetheless, there are case reports demonstrating an allergic reaction to α-parvalbumin [21,22]. Out of all fish molecules evaluated in our study, the lowest sIgE rates were found in thornback ray α-parvalbumin. A study by Kiewiet et al. showed cod parvalbumin (Gad c 1) sensitization rates of 3.2% in British 4 year-olds [7]. However, other study cohorts (Spanish, Italian, Swedish, and Norwegian) in that study showed sensitization rates of 0.1–0.7%. The authors of that study suggested that this difference between the British cohort and cohorts from other countries may be a reflection of dietary habits. According to a European Market Observatory for Fisheries and Aquaculture Products (EUMOFA) report, the mean fish consumption in the European Union was 25.1 kg per person in 2018, with Spain, Italy, and Sweden showing higher consumption of approximately 45 kg, 28 kg, and 27 kg/person, respectively, whereas Poland has a considerably lower fish consumption of 15 kg/person [23]. Therefore, not only geographical regions but also the differences in dietary habits between countries should be evaluated to better understand and manage fish sensitization.

The observed high rates of shrimp sensitization may be attributed to high shrimp consumption [24]. However, despite geographical similarities, the protein sensitization profile in shrimp allergy shows variations. The major shrimp allergens among Hong Kong subjects were troponin C (Pen m 6) and glycogen phosphorylase (Pen m 14, 47.1%), tropomyosin (Pen m 1, 41.2%), and sarcoplasmic-calcium binding protein (Pen m 4, 35.3%), while those among Thai subjects were Pen m 1 (68.8%), Pen m 6 (50.0%), and fatty acid-binding protein (Pen m 13, 37.5%) [25]. In an Italian study conducted on individuals (aged 2–79 years) with a history of adverse reactions to shrimp, sensitization to Pen m 1 was predominant in the molecular sensitization profile; moreover, hypersensitivity to Pen m 1 was closely associated with an increased risk of a severe reaction to mollusks [26]. Shrimp tropomyosin (Pen m 1) is the leading IgE-binding protein in hypersensitivity to this allergen. Our study showed the highest Pen m 1-sIgE rates (1.86%) and levels (9.98 kU_A_/L) out of all five evaluated shrimp molecules. Shellfish allergy often develops in late childhood or during adolescence [27]. In our study, the rates of sIgE in Pen m 1 and Pen m 2 showed a significant increase between the age groups of 1–5 and 5–13 years, and all evaluated shrimp molecules showed the highest sIgE rates in the age group of 13–18-year-olds. Kiewiet et al. also reported increasing rates of sensitization to Pen m 1 with age in a Norwegian cohort (3.4% in 7–12 year-olds; 3.7% in 15–16 year-olds). Although Pen m 1 sIgE rates were not as high in the Swedish cohort, they still showed a tendency to increase with age [7]. Protein profiles in shrimp with hypersensitivity may differ depending on the population, which is likely due to differences in genetics, environmental exposure, and dietary preferences. Therefore, it is important to determine the molecular profile for individual populations to ensure an optimal protein profile in shrimp allergy diagnostic tests [7,24,26,27].

Our study showed the highest rates of tree nut allergen sIgE to be to hazelnut molecule Cor a 1.0401 (23.77%). Importantly, as many as eight various nut molecules (Cor a 1.0401, Jug r 4, Cor a 9, Jug r 2, Jug r 1, Ana o 3, Cor a 14, and Jug r 6) ranked among the 30 most common food allergen molecules. As in the case of peanuts, there was an observable age-related decrease in positive sIgE reactivity to molecules from the family of storage proteins, whereas the Cor a 1.0401 protein, which is a homolog of Bet v 1, showed an inverse tendency. We observed the highest mean sIgE levels for the hazelnut molecule Jug r 1, and this molecule ranked third in the general ranking of mean sIgE levels among all allergen molecules. One of the possible reasons for such a high Jug r 1-sIgE level may be the effect of antibodies to cross-reactive carbohydrate determinants (anti-CCDs) since this molecule is glycosylated and the ALEX^®^ test contains its natural form. These antibodies may be, in part, blocked by the anti-CCD inhibitor that is routinely used in ALEX^®^ [28]. However, the effect of high CCD-sIgE levels on Jug r 1 results cannot be excluded.

## 5. Conclusions

The three most common allergens in Poland, regardless of whether IgE was assayed against extracts or molecules of food allergens, were peanut, hazel, and apple (in different order depending on the ranking). A detailed analysis of sensitization to the extracts and molecules of main food allergens based on the results of a multiplex ALEX^®^ test demonstrated the sensitization profile in Polish children (including molecular sensitization, particularly the “the Big 8” food allergen molecules), which shows considerable differences in comparison with those in other countries. Serum sIgE analysis of children from all regions of Poland revealed a food allergen molecular sensitization profile that changes with age.

## Figures and Tables

**Table 1:** Characteristics of the study group whose results were analyzed to assess sensitization.

		The Number of Results Analyzed	%
Sex	Female	1575	42%
	Male	2140	58%
Age	Mean	7.0	
	Standard deviation	4.16	
	Percentile 25	4.0	
	Median	6.0	
	Percentile 75	10.0	
	Minimum	0.2	
	Maximum	17.3	
	Up to 12 months old	135	4%
	1–5 years old	1469	40%
	>5–13 years old	1721	46%
	>13–18 years old	390	10%

**Table 2:** Analyses of sIgE, which occur at rates of more than 10% of all evaluated food allergen extracts and molecules.

Place in the Ranking of sIgE Frequency *	Tested Allergen	Protein Family	Allergen Source	Number of all sIgE Determinations **	Number and % of Positive sIgE		Place in the Ranking of sIgE Concentration ***	Mean Concentration of Positive sIgE [kU_A_/L]
**Allergen extracts**								
1	Ara h	–	Peanut (*Arachis hypogaea*)	500	146	29.20%	3	8.54
2	Cor a_hazel	–	Hazel (*Corylus avellana*)	500	141	28.20%	24	4.25
3	Mal d	–	Apple (*Malus domestica*)	500	118	23.60%	14	5.69
4	Gal d_egg white	–	Egg white (*Gallus domesticus*)	3715	653	17.58%	18	5.46
5	Ana o	–	Cashew (*Anacardium occidentale*)	3715	570	15.34%	5	7.44
6	Pis v	–	Pistachio (*Pistacia vera*)	500	76	15.20%	10	6.37
7	Gly m	–	Soy (*Glycine max*)	500	73	14.60%	33	3.76
8	Api g	–	Celery (*Apium graveolens*)	500	72	14.40%	36	3.40
9	Jug r_nut	–	Walnut (*Juglans regia*)	500	70	14.00%	13	5.78
10	Cic a	–	Chickpea (*Cicer arietinus*)	3715	503	13.54%	23	4.37
11	Car i	–	Pecan (*Carya illinoensis*)	3715	478	12.87%	8	6.64
12	Dau c	–	Carrot (*Daucus carota*)	3715	476	12.81%	9	6.49
13	Ses i	–	Sesame (*Sesamum indicum*)	3715	439	11.82%	15	5.68
14	Pru du	–	Almond (*Prunus dulcis*)	3715	420	11.31%	29	4.05
15	Hel a	–	Sunflower seed (*Helianthus annuus*)	3715	404	10.87%	48	2.68
16	Pap s	–	Poppy seed (*Papaver somniferum*)	3715	392	10.55%	34	3.70
17	Bos d	–	Cattle (*Bos domesticus*)	3715	383	10.31%	1	9.86
18	Act d	–	Kiwi (*Actinidia deliciosa*)	621	63	10.14%	46	2.74
**Allergen molecules**								
1	Cor a 1.0401	PR-10	Hazel (*Corylus avellana*)	3715	883	23.77%	6	12.22
2	Mal d 1	PR-10	Apple (*Malus domestica*)	3715	831	22.37%	12	10.89
3	Ara h 8	PR-10	Peanut (*Arachis hypogaea*)	3715	629	16.93%	27	7.99
4	Ara h 1	7/8S Globulin	Peanut (*Arachis hypogaea*)	3715	579	15.59%	20	9.35
5	Gly m 4	PR-10	Soy (*Glycine max*)	3715	564	15.18%	15	9.91
6	Api g 1	PR-10	Celery (*Apium graveolens*)	3715	560	15.07%	21	9.08
7	Dau c 1	PR-10	Carrot (*Daucus carota*)	3715	511	13.76%	22	8.64
8	Jug r 4	11S Globulin	Walnut (*Juglans regia*)	3143	431	13.71%	52	4.05
9	Cor a 9	11S Globulin	Hazel (*Corylus avellana*)	3715	486	13.08%	50	4.49
10	Gal d 2	Ovalbumin	Egg white (*Gallus domesticus*)	3715	483	13.00%	47	4.80
11	Ses i 1	2S Albumin	Sesame (*Sesamum indicum*)	3715	453	12.19%	19	9.39
12	Ara h 2	2S Albumin	Peanut (*Arachis hypogaea*)	3715	449	12.09%	1	15.92
13	Gal d 4	Lysozyme C	Egg white (*Gallus domesticus*)	3715	425	11.44%	38	5.32
14	Gal d 1	Ovomucoid	Egg white (*Gallus domesticus*)	3715	422	11.36%	33	6.36
15	Ara h 3	11S Globulin	Peanut (*Arachis hypogaea*)	3715	410	11.04%	31	6.44
16	Jug r 2	7/8S Globulin	Walnut (*Juglans regia*)	3715	401	10.79%	36	5.85
17	Bos d 8	Casein	Cow’s milk (*Bos domesticus*)	3715	389	10.47%	25	8.24

* Place in the ranking of frequency of positive sIgE (i.e., >0.3 kU_A_/L) when all 95 extracts and 77 molecules were investigated (the whole ranking is presented in Table S1); ** The difference in the number of determinations results from the tests performed—ALEX and ALEX2 (explained in detail in the section on the method); *** Place in the ranking of mean concentrations of positive sIgE when all 95 extracts and 77 molecules were investigated (the whole ranking is presented in Table S1).

**Table 3:** Detailed analysis of the “the Big 8” molecules of food allergens in Polish children.

Place in the Ranking of sIgE Frequency Food Allergen Molecules ^a^	Molecule	Protein Family	Allergen Source	Number of All sIgE Determinations Performed	Number and % of “+” sIgE from All sIgE Determinations Performed		Place in the Ranking of Mean sIgE Concentration of Investigated ^b^	Mean Concentration [kU_A_/L]
***Bos domesticus***								
17	Bos d 8	Casein	Cow’s milk (*Bos domesticus*)	3715	389	10.47%	25	8.24
24	Bos d 5	β-Lactoglobulin	Cow’s milk (*Bos domesticus*)	3715	296	7.97%	35	6.22
25	Bos d 4	α-Lactalbumin	Cow’s milk (*Bos domesticus*)	3715	286	7.70%	32	6.37
44	Bos d 6	Serum Albumin	Beef (*Bos domesticus*)	3715	151	4.06%	40	5.28
***Gallus domesticus***								
10	Gal d 2	Ovalbumin	Egg white (*Gallus domesticus*)	3715	483	13.00%	47	4.80
13	Gal d 4	Lysozyme C	Egg white (*Gallus domesticus*)	3715	425	11.44%	38	5.32
14	Gal d 1	Ovomucoid	Egg white (*Gallus domesticus*)	3715	422	11.36%	33	6.36
22	Gal d 3	Ovotransferrin	Egg white (*Gallus domesticus*)	3715	321	8.64%	46	4.81
48	Gal d 5	Serum Albumin	Egg yolk (*Gallus domesticus*)	3715	118	3.18%	61	3.07
***Arachis hypogaea***								
3	Ara h 8	PR-10	Peanut (*Arachis hypogaea*)	3715	629	16.93%	27	7.99
4	Ara h 1	7/8S Globulin	Peanut (*Arachis hypogaea*)	3715	579	15.59%	20	9.35
12	Ara h 2	2S Albumin	Peanut (*Arachis hypogaea*)	3715	449	12.09%	1	15.92
15	Ara h 3	11S Globulin	Peanut (*Arachis hypogaea*)	3715	410	11.04%	31	6.44
18	Ara h 6	2S Albumin	Peanut (*Arachis hypogaea*)	3715	363	9.77%	2	14.43
39	Ara h 9	nsLTP	Peanut (*Arachis hypogaea*)	3715	167	4.50%	54	3.84
72	Ara h 15	Oleosin	Peanut (*Arachis hypogaea*)	3143	14	0.45%	72	1.00
***Glycine max***								
5	Gly m 4	PR-10	Soy (*Glycine max*)	3715	564	15.18%	15	9.91
20	Gly m 6	11S Globulin	Soy (*Glycine max*)	3715	341	9.18%	48	4.63
58	Gly m 5	7/8S Globulin	Soy (*Glycine max*)	3715	77	2.07%	70	1.92
66	Gly m 8	2S Albumin	Soy (*Glycine max*)	3715	43	1.16%	66	2.39
***Triticum aestivum***								
40	Tri a aA_TI	Alpha-Amylase Trypsin-Inhibitor	Wheat (*Triticum aestivum*)	3143	140	4.45%	37	5.78
50	Tri a 14	nsLTP	Wheat (*Triticum aestivum*)	3143	94	2.99%	30	6.52
55	Tri a Gliadin	Gliadin	Wheat (*Triticum aestivum*)	500	12	2.40%	39	5.31
56	Tri a 19	Omega-5-Gliadin	Wheat (*Triticum aestivum*)	3143	74	2.35%	53	4.02
**Fish**								
37	Sco s 1	β-Parvalbumin	Atlantic mackerel (*Scomber scombrus*)	3143	146	4.65%	12	11.19
38	Cyp c 1	β-Parvalbumin	Carp (*Cyprinus carpio*)	3715	169	4.55%	14	10.10
41	Clu h 1	β-Parvalbumin	Atlantic herring (*Clupea harengus*)	3143	136	4.33%	7	12.07
41	Sal s 1	β-Parvalbumin	Salmon (*Salmo salar*)	3143	136	4.33%	10	11.44
42	Thu a 1	β-Parvalbumin	Tuna (*Thunnus albacares*)	3143	134	4.26%	5	12.26
45	Gad m 1	β-Parvalbumin	Atlantic cod (*Gadus morhua*)	3715	150	4.04%	24	8.31
46	Xip g 1	β-Parvalbumin	Swordfish (*Xiphias gladius*)	3143	118	3.75%	18	9.84
69	Raj c Parvalbumin	α-Parvalbumin	Thornback ray (*Thornback ray*)	3143	20	0.64%	46	4.84
**Crustaceans**								
62	Pen m 1	Tropomyosin	Black tiger shrimp (*Penaeus monodon*)	3715	69	1.86%	15	9.98
64	Pen m 2	Arginine Kinase	Black tiger shrimp (*Penaeus monodon*)	3143	49	1.56%	25	8.27
71	Pen m 3	Myosin light chain	Black tiger shrimp (*Penaeus monodon*)	3143	22	0.70%	62	3.16
71	Pen m 4	Sarcoplasmic Calcium Binding Protein	Black tiger shrimp (*Penaeus monodon*)	3143	22	0.70%	30	7.78
73	Cra c 6	Troponin C	Brown shrimp (*Crangon crangon*)	3143	18	0.57%	19	9.65
**Nuts**								
1	Cor a 1.0401	PR-10	Hazel (*Corylus avellana*)	3715	883	23.77%	6	12.22
3	Cor a 11	7/8S Globulin	Hazel (*Corylus avellana*)	3715	638	17.17%	52	4.33
10	Jug r 4	11S Globulin	Walnut (*Juglans regia*)	3143	431	13.71%	54	4.05
11	Cor a 9	11S Globulin	Hazel (*Corylus avellana*)	3715	486	13.08%	51	4.49
18	Jug r 2	7/8S Globulin	Walnut (*Juglans regia*)	3715	401	10.79%	37	5.85
21	Jug r 1	2S Albumin	Walnut (*Juglans regia*)	3715	354	9.53%	3	14.19
23	Ana o 3	2S Albumin	Cashew (*Anacardium occidentale*)	3715	324	8.72%	8	11.93
25	Cor a 14	2S Albumin	Hazel (*Corylus avellana*)	3715	316	8.51%	17	9.85
28	Jug r 6	7/8S Globulin	Walnut (*Juglans regia*)	3143	229	7.29%	43	5.08
49	Cor a 8	nsLTP	Hazel (*Corylus avellana*)	3715	119	3.20%	65	2.55
56	Ana o 2	11S Globulin	Cashew (*Anacardium occidentale*)	3143	77	2.45%	66	2.51
59	Jug r 3	nsLTP	Walnut (*Juglans regia*)	3143	70	2.23%	67	2.42
68	Ber e 1	2S Albumin	Brazil nut (*Bertholletia excelsa*)	3715	37	1.00%	42	5.27

^a^ Place in the ranking of sIgE frequency of investigated 77 food allergen molecules; ^b^ Place in the ranking of average concentration sIgE of investigated 77 food allergen molecules.

## Data Availability

Data supporting reports are available as additional appendices in Tables S1–S3 and from the corresponding.

## Supplementary Materials

The following are available online at https://www.mdpi.com/1422-0067/25/2/825/s1.
