# Supplementary material for: The Sensitization Profile for Selected Food Allergens in Polish Children Assessed with the Use of a Precision Allergy Molecular Diagnostic Technique
Source: Int J Mol Sci. 2024 Jan 9;25(2):825. doi: 10.3390/ijms25020825 (PMC10815771; doi:10.3390/ijms25020825)
Supplement: Supplementary file 1 [file ijms-25-00825-s001.zip › Table S1 The entire ranking of sIgE rates in response to all analyzed food allergen extracts.pdf]

Table A1: The entire ranking of sIgE rates in response to all analyzed food allergen extracts

Place in the ranking of sIgE frequency of investigated 96 food allergen extracts	Tested allergen	Allergen source	Number of all sIgE determinations performed	Number and % of „+” sIgE from all sIgE deter-minations per-formed		Place in the ranking of sIgE frequency of investigated 96 food allergen extracts	Mean concentration [kU/\l]
1	Ara h	Peanut (*Arachis hypogaea*)	500	146	29,20%	3	8,54
2	Cor a hazel	Hazel (*Corylus avellana*)	500	141	28,20%	24	4,25
3	Mal d	Apple (*Malus domestica*)	500	118	23,60%	14	5,69
4	Gal d białko jaja kurzego	Egg white (*Gallus domesticus*)	3715	653	17,58%	18	5,46
5	Ana o	Cashew (*Anacardium occidentale*)	3715	570	15,34%	5	7,44
6	Pis v	Pistachio (*Pistacia vera*)	500	76	15,20%	10	6,37
7	Gly m	Soy (*Glycine max*)	500	73	14,60%	33	3,76
8	Api g	Celery (*Apium graveolens*)	500	72	14,40%	36	3,40
9	Jug r nut	Walnut (*Juglans regia*)	500	70	14,00%	13	5,78
10	Cic a	Chickpea (*Cicer arietinus*)	3715	503	13,54%	23	4,37
11	Car i	Pecan (*Carya illinoensis*)	3715	478	12,87%	8	6,64
12	Dau c	Carrot (*Daucus carota*)	3715	476	12,81%	9	6,49
13	Ses i	Sesame (*Sesamum indicum*)	3715	439	11,82%	15	5,68
14	Pru du	Almond (*Prunus dulcis*)	3715	420	11,31%	29	4,05
15	Hel a	Sunflower seed (*Helianthus annuus*)	3715	404	10,87%	48	2,68
16	Pap s	Poppy seed (*Papaver somniferum*)	3715	392	10,55%	34	3,70
17	Bos d	Cattle (*Bos domesticus*)	3715	383	10,31%	1	9,86
18	Act d	Kiwi (*Actinidia deliciosa*)	621	63	10,14%	46	2,74
19	Ber e	Brazil nut (*Bertholletia excelsa*)	3715	364	9,80%	28	4,06
20	Fag e	Buckwheat (*Fagopyrum esculentum*)	3715	358	9,64%	39	3,28
21	Cuc p	Pumpkin seed (*Cucurbita pepo*)	3715	344	9,26%	20	5,17
22	Ovi a milk	Sheep's milk (*Ovis aries*)	3715	320	8,61%	12	5,94
23	Ave s	Oat (*Avena sativa*)	3715	318	8,56%	44	2,95
24	Sol t	Potato (*Solanum tuberosum*)	3715	312	8,40%	41	3,16
25	Pis s	Pea (*Pisum sativum*)	3715	298	8,02%	31	3,88
26	Pru p	Peach (*Prunus persica*)	500	40	8,00%	37	3,37
27	Len c	Lentil (*Lens culinaris*)	3715	295	7,94%	43	2,98
28	Cap h milk	Goat's milk (*Capra hircus*)	3715	289	7,78%	19	5,24
29	Lup a	Lupine seed (*Lupinus albus*)	3715	275	7,40%	27	4,14
30	All s	Garlic (*Allium sativum*)	3715	264	7,11%	55	1,88
31	Pyr c	Pear (*Pyrus communis*)	3715	251	6,76%	52	2,10

32	Mac inte	Macadamia (*Macadamia integrifolia*)	3715	243	6,54%	17	5,47
33	Tri fo	Fenugreek seed (*Trigonella foenum-graecum*)	3143	188	5,98%	21	4,69
34	Che q	Quinoa (*Chenopodium quinoa*)	3715	215	5,79%	64	1,59
35	Gal d żółtko jaja kurzego	Egg yolk (*Gallus domesticus*)	3715	212	5,71%	40	3,26
36	Sec c flour	Rye (*Secale cereale*)	3715	197	5,30%	36	3,40
36	Sin	Mustard (*Brassica / Sinapis spp.*)	3715	197	5,30%	42	3,03
37	Equ c meat	Horse (*Equus caballus*)	1913	99	5,18%	76	1,17
38	Hor v	Barley (*Hordeum vulgare*)	3715	186	5,01%	53	2,04
39	Tri a	Wheat (*Triticum aestivum*)	500	23	4,60%	25	4,24
40	Tri s	Spelt (*Triticum spelta*)	3715	167	4,50%	38	3,32
41	Gad m	Atlantic cod (*Gadus morhua*)	3715	157	4,23%	6	7,08
42	Bra o	Cabbage (*Brassica oleracea*)	500	21	4,20%	54	2,01
43	Ost e	Oyster (*Ostrea edulis*)	3715	153	4,12%	50	2,35
44	Pha v	White beans (*Phaseolus vulgaris*)	3715	141	3,80%	81	1,01
45	Aga b	White mushroom (*Agaricus hortensis/bisporus*)	500	18	3,60%	56	0,45
46	Sola l	Tomato (*Solanum lycopersicum*)	3715	133	3,58%	90	1,87
47	Pers a	Avocado (*Persea americana*)	3715	127	3,42%	62	1,63
48	Ole fruit	Olive (*Olea Europaea*)	500	17	3,40%	60	1,70
48	Pru do	Plum (*Prunus domestica*)	500	17	3,40%	71	1,28
49	Rub i	Raspberry (*Rubus idaeus*)	500	16	3,20%	51	2,24
50	Cam d	Camel's milk (*Camelus dromedarius*)	3715	111	2,99%	32	3,78
51	Cuc m	Muskmelon (*Cucumis melo*)	500	14	2,80%	74	1,35
52	Pim a	Anise (*Pimpinella anisum*)	3715	103	2,77%	70	1,22
53	Pan m	Millet (*Panicum miliaceum*)	3715	102	2,75%	61	1,64
54	Cap a	Paprika (*Capsicum annuum*)	3715	101	2,72%	58	1,84
55	Clu h	Atlantic herring (*Clupea harengus*)	3143	84	2,67%	22	4,42
56	Bos d nabłonek	Cattle's epithelia (*Bos domesticus*)	500	13	2,60%	63	1,60
57	Ovi a meat	Lamb (*Ovis aries*)	3715	94	2,53%	30	4,03
58	Lol spp.	Squid (*Loligo spp.*)	3715	86	2,31%	26	4,19
59	Myt e	Common mussel (*Mytilus edulis*)	3715	83	2,23%	45	2,77
60	Car p	Papaya (*Carica papaya*)	3715	82	2,21%	49	2,43
60	Hom g	Lobster (*Homarus gammarus*)	3715	82	2,21%	16	5,65
61	Car c	Caraway (*Carum carvi*)	3715	78	2,10%	68	1,41
62	Sal s	Salmon (*Salmo salar*)	3715	74	1,99%	35	3,63
63	Fic c	Fig (*Ficus carica*)	3715	69	1,86%	66	1,46
63	Zea m	Corn, cereal (*Zea mays*)	3715	69	1,86%	65	1,47
64	Ory s	Rice (*Oryza sativa*)	3715	62	1,67%	75	1,18
64	Vac m	Blueberry (*Vaccinium myrtillus*)	3715	62	1,67%	88	0,75
65	Ory meat	Rabbit (*Oryctolagus spp.*)	3715	60	1,62%	59	1,83
66	Equ c milk	Mare's milk (*Equus caballus*)	3715	58	1,56%	45	2,77
67	Pru av	Cherry (*Prunus spp.*)	3715	57	1,53%	68	1,41

68	All c	Onion (*Allium cepa*)	3715	56	1,51%	77	1,15
69	Chi spp.	Crab (*Chionoecetes spp.*)	3715	52	1,40%	4	8,38
70	Bro pa	Paper mulberry (*Broussonetia papyrifera*)	3143	43	1,37%	87	0,76
71	Pan b	Northern prawn (*Pandalus borealis*)	3715	50	1,35%	2	6,17
72	Gal d mięso	Chicken (*Gallus domesticus*)	3715	46	1,24%	67	1,45
72	Lit s	*aeus setiferus, Farfantepenaeus aztecus, Farfant*	3715	46	1,24%	11	8,55
72	Mel g	Turkey (*Meleagris gallopavo*)	3715	46	1,24%	57	1,86
73	Mus a	Banana (*Musa acuminata*)	3715	37	1,00%	82	0,99
74	Sus d meat	Pig (*Sus domesticus*)	3715	36	0,97%	70	1,35
75	Thu a	Tuna (*Thunnus albacares*)	3715	35	0,94%	78	1,11
76	Sco s	Atlantic mackerel (*Scomber scombrus*)	3143	29	0,92%	84	0,92
76	Che a	Lamb's quarter (*Chenopodium album*)	3715	34	0,92%	47	2,71
77	Pet c	Parsley (*Petroselinum crispum*)	3715	30	0,81%	72	1,27
77	Rud spp.	Venus clam (*Ruditapes spp.*)	3715	30	0,81%	7	6,89
78	Lit c	Lychee (*Litchi chinensis*)	500	4	0,80%	79	0,49
79	Cit s	Orange (*Citrus sinensis*)	3715	28	0,75%	69	1,08
79	Pec spp.	Scallop (*Pecten spp.*)	3715	28	0,75%	89	1,39
80	Mor r	Mulberry tree (*Morus rubra*)	3715	26	0,70%	85	0,89
81	Man i	Mango (*Mangifera indica*)	3715	23	0,62%	83	0,98
82	Lac s	Lettuce (*Lactuca sativa*)	500	3	0,60%	73	1,24
83	Fra a	Strawberry (*Fragaria ananassa*)	500	2	0,40%	91	0,40
84	Ori v	Oregano (*Origanum vulgare*)	3715	11	0,30%	80	1,05
85	Raj c	Thornback ray (*Raja clavata*)	3143	5	0,16%	86	0,78
