# Supplementary material for: The Sensitization Profile for Selected Food Allergens in Polish Children Assessed with the Use of a Precision Allergy Molecular Diagnostic Technique
Source: Int J Mol Sci. 2024 Jan 9;25(2):825. doi: 10.3390/ijms25020825 (PMC10815771; doi:10.3390/ijms25020825)
Supplement: Supplementary file 1 [file ijms-25-00825-s001.zip › Table S2 The entire ranking of sIgE rates in response to all analyzed food allergen molecules.pdf]

Table A2: The entire ranking of sIgE rates in response to all analyzed food allergen molecules

Place in the ranking of sIgE frequency of investigated 80 food allergen molecules	Molecule	Protein family	Allergen source	Number of all sIgE determinations performed	Number and % of „+” sIgE from all sIgE determinations performed		Place in the ranking of sIgE frequency of investigated 77 food allergen molecules	Mean concentration [kU/\l]
1	rCor a 1.0401	PR-10	Hazel (*Corylus avellana*)	3715	883	23,77%	6	12,22
2	rMal d 1	PR-10	Apple (*Malus domestica*)	3715	831	22,37%	12	10,89
3	rAra h 8	PR-10	Peanut (*Arachis hypogaea*)	3715	629	16,93%	27	7,99
4	nAra h 1	7/8S Globulin	Peanut (*Arachis hypogaea*)	3715	579	15,59%	20	9,35
5	rGly m 4	PR-10	Soy (*Glycine max*)	3715	564	15,18%	15	9,91
6	rApi g 1	PR-10	Celery (*Apium graveolens*)	3715	560	15,07%	21	9,08
7	rDau c 1	PR-10	Carrot (*Daucus carota*)	3715	511	13,76%	22	8,64
8	nJug r 4	11S Globulin	Walnut (*Juglans regia*)	3143	431	13,71%	52	4,05
9	nCor a 9	11S Globulin	Hazel (*Corylus avellana*)	3715	486	13,08%	50	4,49
10	nGal d 2	Ovalbumin	Egg white (*Gallus domesticus*)	3715	483	13,00%	47	4,80
11	nSes i 1	2S Albumin	Sesame (*Sesamum indicum*)	3715	453	12,19%	19	9,39
12	rAra h 2	2S Albumin	Peanut (*Arachis hypogaea*)	3715	449	12,09%	1	15,92
13	nGal d 4	Lysozyme C	Egg white (*Gallus domesticus*)	3715	425	11,44%	38	5,32
14	nGal d 1	Ovomucoid	Egg white (*Gallus domesticus*)	3715	422	11,36%	33	6,36
15	nAra h 3	11S Globulin	Peanut (*Arachis hypogaea*)	3715	410	11,04%	31	6,44
16	nJug r 2	7/8S Globulin	Walnut (*Juglans regia*)	3715	401	10,79%	36	5,85
17	nBos d 8	Casein	Cow's milk (*Bos domesticus*)	3715	389	10,47%	25	8,24
18	nAra h 6	2S Albumin	Peanut (*Arachis hypogaea*)	3715	363	9,77%	2	14,43
19	nJug r 1	2S Albumin	Walnut (*Juglans regia*)	3715	354	9,53%	3	14,19
20	nGly m 6	11S Globulin	Soy (*Glycine max*)	3715	341	9,18%	48	4,63
21	rAna o 3	2S Albumin	Cashew (*Anacardium occidentale*)	3715	324	8,72%	8	11,93
22	nGal d 3	Ovotransferrin	Egg white (*Gallus domesticus*)	3715	321	8,64%	46	4,81
23	nCor a 14	2S Albumin	Hazel (*Corylus avellana*)	3715	316	8,51%	16	9,85
24	nBos d 5	β-Lactoglobulin	Cow's milk (*Bos domesticus*)	3715	296	7,97%	35	6,22

25	nBos d 4	α-Lactalbumin	Cow's milk (*Bos domesticus*)	3715	286	7,70%	32	6,37
26	nJug r 6	7/8S Globulin	Walnut (*Juglans regia*)	3143	229	7,29%	42	5,08
27	rPis v 1	2S Albumin	Pistachio (*Pistacia vera*)	3143	228	7,25%	9	11,50
28	rPru p 3	nsLTP	Peach (*Prunus persica*)	3715	241	6,49%	55	3,72
29	rCuc m 2	Profilin	Muskmelon (*Cucumis melo*)	3143	195	6,20%	4	12,92
30	nAct d 1	Cysteine Protease	Kiwi (*Actinidia deliciosa*)	3715	217	5,84%	34	6,28
31	rSus d 1	Serum Albumin	Pig (*Sus domesticus*)	3143	170	5,41%	28	7,95
32	rMal d 3	nsLTP	Apple (*Malus domestica*)	3715	187	5,03%	59	3,22
33	nSin a 1	2S Albumin	Mustard (*Brassica / Sinapis spp.*)	3715	184	4,95%	44	5,00
34	rZea m 14	nsLTP	Corn, cereal (*Zea mays*)	3143	154	4,90%	58	3,26
35	nPis v 2	11S Globulin	Pistachio (*Pistacia vera*)	3143	148	4,71%	57	3,41
36	nPis v 3	7S wicilina	Pistachio (*Pistacia vera*)	3143	147	4,68%	50	4,49
37	rSco s 1	β-Parvalbumin	tlantic mackerel (*Scomber scombru*	3143	146	4,65%	11	11,19
38	rCyp c 1	β-Parvalbumin	Carp (*Cyprinus carpio*)	3715	169	4,55%	13	10,10
39	rAra h 9	nsLTP	Peanut (*Arachis hypogaea*)	3715	167	4,50%	54	3,84
40	nTri a aA_TI	ha-Amylase Trypsin-Inhib	Wheat (*Triticum aestivum*)	3143	140	4,45%	37	5,78
41	rClu h 1	β-Parvalbumin	Atlantic herring (*Clupea harengus*)	3143	136	4,33%	7	12,07
41	rSal s 1	β-Parvalbumin	Salmon (*Salmo salar*)	3143	136	4,33%	10	11,44
42	rThu a 1	β-Parvalbumin	Tuna (*Thunnus albacares*)	3143	134	4,26%	5	12,26
44	nAct d 10	nsLTP	Kiwi (*Actinidia deliciosa*)	3715	151	4,06%	61	3,07
44	nBos d 6	Serum Albumin	Beef (*Bos domesticus*)	3715	151	4,06%	40	5,28
45	nGad m 1	β-Parvalbumin	Atlantic cod (*Gadus morhua*)	3715	150	4,04%	23	8,31
46	rXip g 1	β-Parvalbumin	Swordfish (*Xiphias gladius*)	3143	118	3,75%	17	9,84
47	rCor a 8	nsLTP	Hazel (*Corylus avellana*)	3715	119	3,20%	63	2,55
48	nGal d 5	Serum Albumin	Egg yolk (*Gallus domesticus*)	3715	118	3,18%	61	3,07
49	nVit v 1	nsLTP	Grape (*Vitis vinifera*)	3715	117	3,15%	59	3,22
50	rTri a 14	nsLTP	Wheat (*Triticum aestivum*)	3143	94	2,99%	30	6,52
51	nPap s 2S	2S Albumin	Poppy seed (*Papaver somniferum*)	3715	108	2,91%	49	4,59
52	nMac i 2S	2S Albumin	Iacadamia (*Macadamia integrifolia*	3715	97	2,61%	43	5,02
53	rApi g 2	nsLTP	Celery (*Apium graveolens*)	3715	95	2,56%	69	1,93
54	rAna o 2	11S Globulin	Cashew (*Anacardium occidentale*)	3143	77	2,45%	64	2,51
55	nTri a Gliadin	Gliadin	Wheat (*Triticum aestivum*)	500	12	2,40%	39	5,31
56	rTri a 19	Omega-5-Gliadin	Wheat (*Triticum aestivum*)	3143	74	2,35%	53	4,02
57	rJug r 3	nsLTP	Walnut (*Juglans regia*)	3143	70	2,23%	65	2,42
58	rGly m 5	7/8S Globulin	Soy (*Glycine max*)	3715	77	2,07%	70	1,92
59	nAct d 2	TLP	Kiwi (*Actinidia deliciosa*)	3715	76	2,05%	68	2,10

59	rAni s 3	Tropomyosin	Anisakis simplex (*Anisakis simplex*)	3715	76	2,05%	26	8,08
60	nPen m 1	Tropomyosin	ack tiger shrimp (*Penaeus monodo*	3715	69	1,86%	14	9,98
61	rApi g 6	nsLTP	Celery (*Apium graveolens*)	3715	66	1,78%	56	3,42
63	rPen m 2	Arginine Kinase	ack tiger shrimp (*Penaeus monodo*	3143	49	1,56%	24	8,27
64	nAct d 5	Kiwellin	Kiwi (*Actinidia deliciosa*)	3715	57	1,53%	62	2,73
65	nSola l 6	nsLTP	Tomato (*Solanum lycopersicum*)	3715	52	1,40%	51	4,28
66	nGly m 8	2S Albumin	Soy (*Glycine max*)	3715	43	1,16%	66	2,39
67	nBer e 1	2S Albumin	Brazil nut (*Bertholletia excelsa*)	3715	37	1,00%	41	5,27
68	nFag e 2	2S Albumin	3uckwheat (Fagopyrum esculentum	3715	36	0,97%	67	2,28
69	rPen m 3	Myosin light chain	ack tiger shrimp (*Penaeus monodo*	3143	22	0,70%	60	3,16
69	rPen m 4	lasmic Calcium Binding F	ack tiger shrimp (*Penaeus monodo*	3143	22	0,70%	29	7,78
70	rRaj c	α-Parvalbumin	Thornback ray (*Thornback ray*)	3143	20	0,64%	45	4,84
71	rCra c 6	Troponin C	Brown shrimp (*Crangon crangon*)	3143	18	0,57%	18	9,65
72	rAra h 15	Oleosin	Peanut (*Arachis hypogaea*)	3143	14	0,45%	72	1,00
73	rChe a 1	Ole e 1-Family	amb's quarter (*Chenopodium albun*	3715	16	0,43%	71	1,66
74	rAni s 1	itz Serine Protease Inhib	Anisakis simplex (*Anisakis simplex*)	3715	11	0,30%	74	0,78
75	nMal d 2	TLP	Apple (*Malus domestica*)	3715	10	0,27%	73	0,99
