# Supplementary material for: The Sensitization Profile for Selected Food Allergens in Polish Children Assessed with the Use of a Precision Allergy Molecular Diagnostic Technique
Source: Int J Mol Sci. 2024 Jan 9;25(2):825. doi: 10.3390/ijms25020825 (PMC10815771; doi:10.3390/ijms25020825)
Supplement: Supplementary file 1 [file ijms-25-00825-s001.zip › Table S3 sIgE against the Big 8 food allergen molecules stratified by age.pdf]

Table A3: sIgE against "the Big 8" food allergen molecules stratified by age

		Age: Up to 12 months N	Up to 12 months %	Age: 1-5 years old N	1-5 years old %	Chi-square test χ^2	Chi-square test p
**Bos domesticus**							
nBos d 4	Positive	34	25,20%	152	10,30%	27	<0,001*
	Total	135	100,00%	1469	100,00%		
nBos d 5	Positive	32	23,70%	157	10,70%	20	<0,001*
	Total	135	100,00%	1469	100,00%		
nBos d 6	Pozytywne	11	8,10%	64	4,40%	4	0,046*
	Total	135	100,00%	1469	100,00%		
nBos d 8	Positive	41	30,40%	202	13,80%	27	<0,001*
	Total	135	100,00%	1469	100,00%		
**Gallus domesticus**							
nGal d 1	Positive	37	27,40%	216	14,70%	15	<0,001*
	Total	135	100,00%	1469	100,00%		
nGal d 2	Positive	50	37,00%	250	17,00%	33	<0,001*
	Total	135	100,00%	1469	100,00%		
nGal d 3	Positive	19	14,10%	161	11,00%	1,2	0,273
	Total	135	100,00%	1469	100,00%		
nGal d 4	Positive	41	30,40%	236	16,10%	18	<0,001*
	Total	135	100,00%	1469	100,00%		
nGal d 5	Positive	6	4,40%	49	3,30%	0,5	0,498[a]
	Total	135	100,00%	1469	100,00%		
**Arachis hypogaea**							
nAra h 1	Positive	36	26,70%	290	19,70%	3,7	0,056
	Total	135	100,00%	1469	100,00%		
rAra h 2	Positive	20	14,80%	229	15,60%	0,1	0,812
	Total	135	100,00%	1469	100,00%		
nAra h 3	Positive	19	14,10%	194	13,20%	0,1	0,776
	Total	135	100,00%	1469	100,00%		
nAra h 6	Positive	13	9,60%	161	11,00%	0,2	0,634

		Age: 1-5 years old N	1-5 years old %	Age: 5-13 years old N	5-13 years old %	Chi-square test χ^2	Chi-square test p
**Bos domesticus**							
nBos d 4	Positive	152	10,30%	94	5,50%	27	<0,001*
	Total	1469	100,00%	1721	100,00%		
nBos d 5	Positive	157	10,70%	100	5,80%	25	<0,001*
	Total	1469	100,00%	1721	100,00%		
nBos d 6	Pozytywne	64	4,40%	66	3,80%	0,6	0,458
	Total	1469	100,00%	1721	100,00%		
nBos d 8	Positive	202	13,80%	133	7,70%	31	<0,001*
	Total	1469	100,00%	1721	100,00%		
**Gallus domesticus**							
nGal d 1	Positive	216	14,70%	150	8,70%	28	<0,001*
	Total	1469	100,00%	1721	100,00%		
nGal d 2	Positive	250	17,00%	165	9,60%	39	<0,001*
	Total	1469	100,00%	1721	100,00%		
nGal d 3	Positive	161	11,00%	129	7,50%	12	0,001*
	Total	1469	100,00%	1721	100,00%		
nGal d 4	Positive	236	16,10%	129	7,50%	57	<0,001*
	Total	1469	100,00%	1721	100,00%		
nGal d 5	Positive	49	3,30%	57	3,30%	0	0,97
	Total	1469	100,00%	1721	100,00%		
**Arachis hypogaea**							
nAra h 1	Positive	290	19,70%	229	13,30%	24	<0,001*
	Total	1469	100,00%	1721	100,00%		
rAra h 2	Positive	229	15,60%	182	10,60%	18	<0,001*
	Total	1469	100,00%	1721	100,00%		
nAra h 3	Positive	194	13,20%	176	10,20%	6,9	0,009*
	Total	1469	100,00%	1721	100,00%		
nAra h 6	Positive	161	11,00%	170	9,90%	1	0,318

		Age: 5-13 years old N	5-13 years old %	Age: 13-18 years old N	13-18 years old %	Chi-square test χ^2	Chi-square test p
**Bos domesticus**							
nBos d 4	Positive	94	5,50%	6	1,50%	10,9	0,001*
	Total	1721	100,00%	390	100,00%		
nBos d 5	Positive	100	5,80%	7	1,80%	10,7	0,001*
	Total	1721	100,00%	390	100,00%		
nBos d 6	Pozytywne	66	3,80%	10	2,60%	1,48	0,224
	Total	1721	100,00%	390	100,00%		
nBos d 8	Positive	133	7,70%	13	3,30%	9,54	0,002*
	Total	1721	100,00%	390	100,00%		
**Gallus domesticus**							
nGal d 1	Positive	150	8,70%	19	4,90%	6,38	0,012*
	Total	1721	100,00%	390	100,00%		
nGal d 2	Positive	165	9,60%	18	4,60%	9,93	0,002*
	Total	1721	100,00%	390	100,00%		
nGal d 3	Positive	129	7,50%	12	3,10%	9,96	0,002*
	Total	1721	100,00%	390	100,00%		
nGal d 4	Positive	129	7,50%	19	4,90%	3,36	0,067
	Total	1721	100,00%	390	100,00%		
nGal d 5	Positive	57	3,30%	6	1,50%	3,45	0,063
	Total	1721	100,00%	390	100,00%		
**Arachis hypogaea**							
nAra h 1	Positive	229	13,30%	24	6,20%	15,4	<0,001*
	Total	1721	100,00%	390	100,00%		
rAra h 2	Positive	182	10,60%	18	4,60%	13,2	<0,001*
	Total	1721	100,00%	390	100,00%		
nAra h 3	Positive	176	10,20%	21	5,40%	8,81	0,003*
	Total	1721	100,00%	390	100,00%		
nAra h 6	Positive	170	9,90%	19	4,90%	9,78	0,002*

[illegible]	Total	135	100,00%	1469	100,00%	[illegible]	[illegible]	1469	100,00%	1721	100,00%	[illegible]	[illegible]	1721	100,00%	390	100,00%	[illegible]	[illegible]
rAra h 8	Positive	0	0,00%	114	7,80%	11	0,001*	114	7,80%	394	22,90%	136	<0,001*	394	22,90%	121	31,00%	11,4	0,001*
	Total	135	100,00%	1469	100,00%			1469	100,00%	1721	100,00%			1721	100,00%	390	100,00%		
rAra h 9	Positive	2	1,50%	62	4,20%	2,4	0,12	62	4,20%	89	5,20%	1,6	0,207	89	5,20%	14	3,60%	1,71	0,19
	Total	135	100,00%	1469	100,00%			1469	100,00%	1721	100,00%			1721	100,00%	390	100,00%		
rAra h 15	Positive	0	0,00%	6	0,50%	0,6	0,435[a,b]	6	0,50%	5	0,40%	0,1	0,724	5	0,40%	3	0,90%	1,73	0,188[a]
	Total	134	100,00%	1322	100,00%			1322	100,00%	1363	100,00%			1363	100,00%	324	100,00%		
								Glycine max											
rGly m 4	Positive	0	0,00%	102	6,90%	10	0,002*	102	6,90%	363	21,10%	127	<0,001*	363	21,10%	99	25,40%	3,43	0,064
	Total	135	100,00%	1469	100,00%			1469	100,00%	1721	100,00%			1721	100,00%	390	100,00%		
rGly m 5	Positive	2	1,50%	28	1,90%	0,1	0,727[a]	28	1,90%	43	2,50%	1,3	0,258	43	2,50%	4	1,00%	3,17	0,075
	Total	135	100,00%	1469	100,00%			1469	100,00%	1721	100,00%			1721	100,00%	390	100,00%		
nGly m 6	Positive	17	12,60%	161	11,00%	0,3	0,563	161	11,00%	148	8,60%	5,1	0,025*	148	8,60%	15	3,80%	10,1	0,001*
	Total	135	100,00%	1469	100,00%			1469	100,00%	1721	100,00%			1721	100,00%	390	100,00%		
nGly m 8	Positive	0	0,00%	14	1,00%	1,3	0,255[a]	14	1,00%	25	1,50%	1,6	0,201	25	1,50%	4	1,00%	0,43	0,513
	Total	135	100,00%	1469	100,00%			1469	100,00%	1721	100,00%			1721	100,00%	390	100,00%		
								Triticum aestivum											
nTri a aA_TI	Positive	29	21,60%	61	4,60%	61	<0,001*	61	4,60%	45	3,30%	3,1	0,081	45	3,30%	5	1,50%	2,81	0,093
	Total	134	100,00%	1322	100,00%			1322	100,00%	1363	100,00%			1363	100,00%	324	100,00%		
rTri a 14	Positive	8	6,00%	45	3,40%	2,3	0,131[a]	45	3,40%	36	2,60%	1,3	0,248	36	2,60%	5	1,50%	1,33	0,249
	Total	134	100,00%	1322	100,00%			1322	100,00%	1363	100,00%			1363	100,00%	324	100,00%		
rTri a 19	Positive	24	17,90%	33	2,50%	77	<0,001*	33	2,50%	15	1,10%	7,5	0,006*	15	1,10%	2	0,60%	0,61	0,434[a]
	Total	134	100,00%	1322	100,00%			1322	100,00%	1363	100,00%			1363	100,00%	324	100,00%		
								Fish											
rClu h 1	Positive	4	3,00%	55	4,20%	0,4	0,511[b]	55	4,20%	70	5,10%	1,4	0,23	70	5,10%	7	2,20%	5,32	0,021*
	Total	134	100,00%	1322	100,00%			1322	100,00%	1363	100,00%			1363	100,00%	324	100,00%		
rCyp c 1	Positive	5	3,70%	55	3,70%	0	0,981	55	3,70%	100	5,80%	7,3	0,007*	100	5,80%	9	2,30%	7,97	0,005*
	Total	135	100,00%	1469	100,00%			1469	100,00%	1721	100,00%			1721	100,00%	390	100,00%		
nGad m 1	Positive	2	1,50%	51	3,50%	1,5	0,216[a]	51	3,50%	85	4,90%	4,2	0,041*	85	4,90%	12	3,10%	2,51	0,113
	Total	135	100,00%	1469	100,00%			1469	100,00%	1721	100,00%			1721	100,00%	390	100,00%		
nGad m 2&3	Positive	0	0,00%	11	0,80%	1,1	0,289[a]	11	0,80%	16	1,20%	0,8	0,375	16	1,20%	2	0,60%	0,77	0,381[a]
	Total	134	100,00%	1322	100,00%			1322	100,00%	1363	100,00%			1363	100,00%	324	100,00%		
rRaj c Parvalbu[illegible]	Positive	0	0,00%	7	0,50%	0,7	0,398[a, b]	7	0,50%	12	0,90%	1,2	0,278	12	0,90%	1	0,30%	1,12	0,290[a]

Parvalbumin	Total	134	100,00%	1322	100,00%	0,7	0,398	1322	100,00%	1363	100,00%	1,2	[illegible]	1363	100,00%	324	100,00%	1,12	0,290
rSal s 1	Positive	3	2,20%	57	4,30%	1,3	0,25	57	4,30%	70	5,10%	1	0,315	70	5,10%	6	1,90%	6,56	0,010*
	Total	134	100,00%	1322	100,00%			1322	100,00%	1363	100,00%			1363	100,00%	324	100,00%		
rSco s 1	Positive	4	3,00%	56	4,20%	0,5	0,488	56	4,20%	80	5,90%	3,7	0,054	80	5,90%	6	1,90%	8,73	0,003*
	Total	134	100,00%	1322	100,00%			1322	100,00%	1363	100,00%			1363	100,00%	324	100,00%		
rThu a 1	Positive	3	2,20%	54	4,10%	1,1	0,294	54	4,10%	70	5,10%	1,7	0,195	70	5,10%	7	2,20%	5,32	0,021*
	Total	134	100,00%	1322	100,00%			1322	100,00%	1363	100,00%			1363	100,00%	324	100,00%		
rXip g 1	Positive	4	3,00%	44	3,30%	0	0,832[a]	44	3,30%	63	4,60%	2,9	0,087	63	4,60%	7	2,20%	3,99	0,046*
	Total	134	100,00%	1322	100,00%			1322	100,00%	1363	100,00%			1363	100,00%	324	100,00%		
Crustaceans																			
nPen m 1	Positive	2	1,50%	17	1,20%	0,1	0,739[a]	17	1,20%	40	2,30%	6,2	0,013*	40	2,30%	10	2,60%	0,08	0,779
	Total	135	100,00%	1469	100,00%			1469	100,00%	1721	100,00%			1721	100,00%	390	100,00%		
rPen m 2	Positive	0	0,00%	5	0,40%	0,5	0,476[a,b]	5	0,40%	32	2,30%	19	<0,001*	32	2,30%	12	3,70%	1,89	0,169
	Total	134	100,00%	1322	100,00%			1322	100,00%	1363	100,00%			1363	100,00%	324	100,00%		
rPen m 3	Positive	1	0,70%	7	0,50%	0,1	0,746[a,b]	7	0,50%	10	0,70%	0,4	0,505	10	0,70%	4	1,20%	0,8	0,372[a]
	Total	134	100,00%	1322	100,00%			1322	100,00%	1363	100,00%			1363	100,00%	324	100,00%		
rPen m 4	Positive	1	0,70%	8	0,60%	0	0,843[a,b]	8	0,60%	10	0,70%	0,2	0,683	10	0,70%	3	0,90%	0,13	0,722[a]
	Total	134	100,00%	1322	100,00%			1322	100,00%	1363	100,00%			1363	100,00%	324	100,00%		
rCra c 6	Positive	0	0,00%	1	0,10%	0,1	0,750[a,b]	1	0,10%	12	0,90%	9	0,003*	12	0,90%	5	1,50%	1,15	0,283[a]
	Total	134	100,00%	1322	100,00%			1322	100,00%	1363	100,00%			1363	100,00%	324	100,00%		
Nuts																			
rCor a 1.0401	Positive	0	0,00%	177	12,00%	18	<0,001*	177	12,00%	549	31,90%	178	<0,001*	549	31,90%	157	40,30%	9,97	0,002*
	Total	135	100,00%	1469	100,00%			1469	100,00%	1721	100,00%			1721	100,00%	390	100,00%		
rCor a 8	Positive	1	0,70%	46	3,10%	2,5	0,115[a]	46	3,10%	65	3,80%	1	0,321	65	3,80%	7	1,80%	3,79	0,052
	Total	135	100,00%	1469	100,00%			1469	100,00%	1721	100,00%			1721	100,00%	390	100,00%		
nCor a 9	Positive	41	30,40%	261	17,80%	13	<0,001*	261	17,80%	169	9,80%	43	<0,001*	169	9,80%	15	3,80%	14,3	<0,001*
	Total	135	100,00%	1469	100,00%			1469	100,00%	1721	100,00%			1721	100,00%	390	100,00%		
nCor a 11	Positive	9	56,30%	48	28,60%	5,2	0,022[a],*	48	28,60%	41	24,80%	0,6	0,443	41	24,80%	1	2,30%	11	0,001*
	Total	16	100,00%	168	100,00%			168	100,00%	165	100,00%			165	100,00%	44	100,00%		
nCor a 14	Positive	7	5,20%	160	10,90%	4,3	0,038*	160	10,90%	136	7,90%	8,4	0,004*	136	7,90%	13	3,30%	10,1	0,001*
	Total	135	100,00%	1469	100,00%			1469	100,00%	1721	100,00%			1721	100,00%	390	100,00%		
nJug r 1	Positive	7	5,20%	170	11,60%	5,1	0,023*	170	11,60%	158	9,20%	4,9	0,027*	158	9,20%	19	4,90%	7,68	0,006*
	Total	135	100,00%	1469	100,00%			1469	100,00%	1721	100,00%			1721	100,00%	390	100,00%		

nJug r 2	Positive	6	4,40%	171	11,60%	6,5	0,011*	171	11,60%	195	11,30%	0,1	0,784	195	11,30%	29	7,40%	5,08	0,024*
	Total	135	100,00%	1469	100,00%			1469	100,00%	1721	100,00%			1721	100,00%	390	100,00%		
rJug r 3	Positive	1	0,70%	24	1,80%	0,8	0,364[a]	24	1,80%	39	2,90%	3,2	0,073	39	2,90%	6	1,90%	1,03	0,311
	Total	134	100,00%	1322	100,00%			1322	100,00%	1363	100,00%			1363	100,00%	324	100,00%		
nJug r 4	Positive	35	26,10%	239	18,10%	5,2	0,023*	239	18,10%	141	10,30%	33	<0,001*	141	10,30%	16	4,90%	9,07	0,003*
	Total	134	100,00%	1322	100,00%			1322	100,00%	1363	100,00%			1363	100,00%	324	100,00%		
nJug r 6	Positive	5	3,70%	100	7,60%	2,7	0,102	100	7,60%	110	8,10%	0,2	0,625	110	8,10%	14	4,30%	5,4	0,020*
	Total	134	100,00%	1322	100,00%			1322	100,00%	1363	100,00%			1363	100,00%	324	100,00%		

*. The chi-square statistic is significant at the 0.05 level.

a. More than 20% of the cells in this sub-table have an expected sample size lower than 5. The results of the chi-square test may not be correct.

b. The minimum expected sample size in cells for this sub-table is less than one. The results of the chi-square test may not be correct.
