# Supplementary figures and images for: The Sensitization Profile for Selected Food Allergens in Polish Children Assessed with the Use of a Precision Allergy Molecular Diagnostic Technique
Source: Int J Mol Sci. 2024 Jan 9;25(2):825. doi: 10.3390/ijms25020825 (PMC10815771; doi:10.3390/ijms25020825)

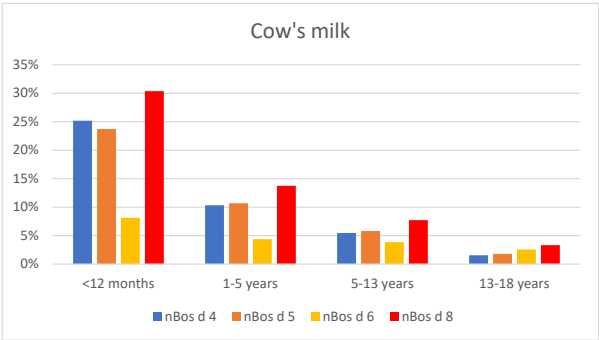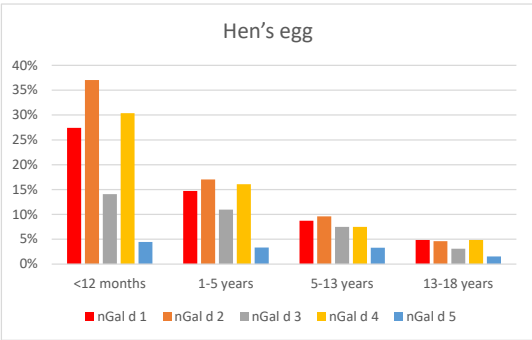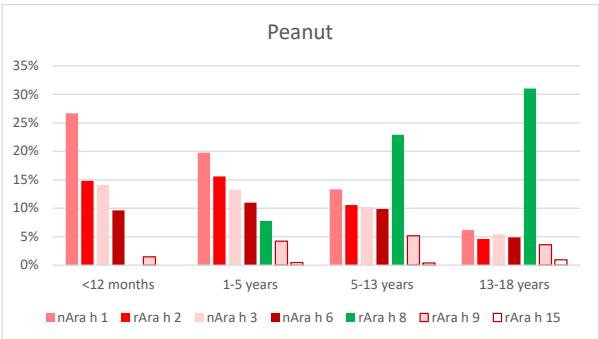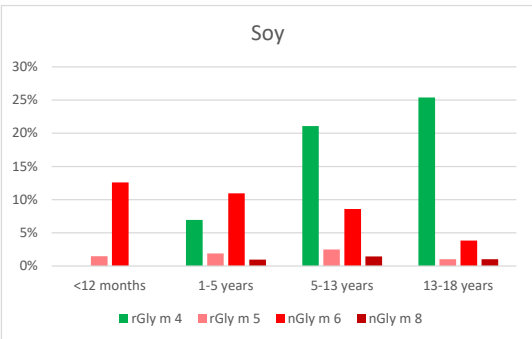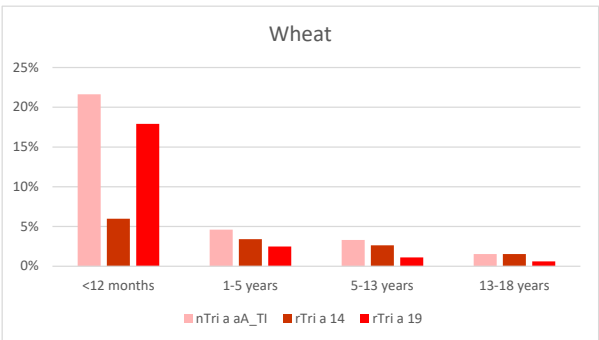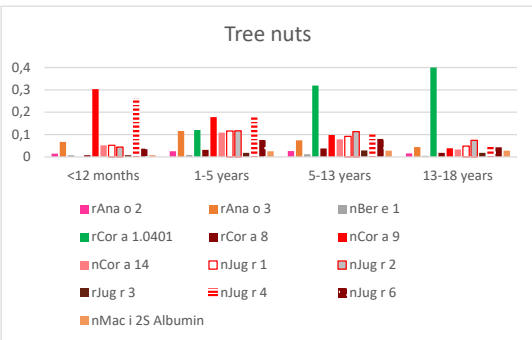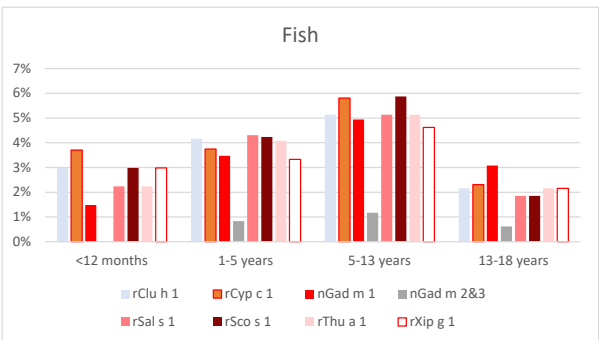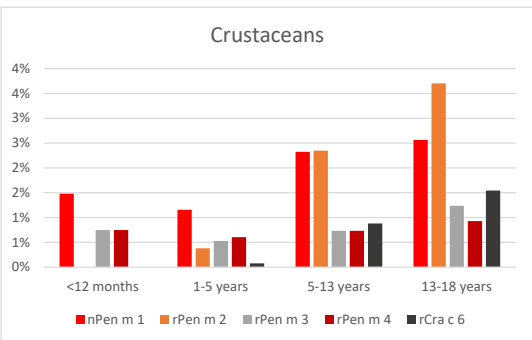

Supplement: Supplementary file 1 [file ijms-25-00825-s001.zip › Figure S1.pdf]
